# The effects of music-based interventions on cognitive function in cognitively normal older adults: a systematic review and meta-analysis

**DOI:** 10.3389/fpsyg.2025.1632873

**Published:** 2025-11-18

**Authors:** LiJuan Tang, Zan Feng, Yunxu Zhang, Feng Tong

**Affiliations:** 1 School of Humanities, Southwest Jiaotong University, Chengdu, China; 2 International School of Law and Society, Sichuan International Studies University, Chongqing, China

**Keywords:** music-based intervention, older adults, cognitive function, systematic review, meta-analysis

## Abstract

**Objective:**

This systematic review and meta-analysis aimed to investigate whether music-based interventions (MBIs) can improve cognitive function in cognitively normal older adults.

**Methods:**

We systematically searched multiple databases through March 2025. Only randomized controlled trials (RCTs) were included. Eligible interventions were structured programs with music as the core component. Participants were cognitively normal adults aged ≥60 years. Primary outcome measures included global cognition, memory, executive function, and attention. A random-effects model meta-analysis was conducted to synthesize effect sizes.

**Results:**

Nine RCTs (total *N* = 625 participants) met the inclusion criteria. Music-based interventions significantly improved global cognition [SMD = 0.31, 95% CI (0.11, 0.52)], memory [SMD = 0.36, 95% CI (0.04, 0.69)], and executive function [SMD = 0.43, 95% CI (0.11, 0.74)] compared to control groups. However, no significant improvement in attention was observed [SMD = −0.12, 95% CI (−0.34, 0.11)].

**Conclusion:**

Music-based interventions have positive effects on global cognition, executive function, and memory in cognitively normal older adults, but there is no evidence of improvement in attention. Larger sample sizes and higher-quality studies are needed to confirm these findings.

## 1 Introduction

Population aging is a prominent global demographic trend. According to projections by the United Nations Population Division, by 2050 the population aged ≥65 years is expected to reach approximately 1.5 billion—about “one in six” people worldwide (United Nations Department of Economic and Social Affairs, Population Division, 2024). Aging is commonly accompanied by declines in cognitive function across multiple domains, including memory, executive function, visuospatial abilities, language, perception, and attention (Harada et al., 2013). Cognitive decline is a risk factor for Alzheimer's disease and diminishes older adults' self-efficacy and quality of life (Aye et al., 2023). The maintenance of cognitive function is therefore a key component of healthy aging. Early, effective training or preventive interventions to slow cognitive decline in older adults are thus of considerable importance for promoting healthy aging.

In recent years, music-based interventions (MBIs) have emerged as an important non-pharmacological approach to health promotion and rehabilitation (McCrary et al., 2022). According to the NIH Music-Based Intervention Toolkit (Edwards et al., 2023) and the *Reporting Guidelines for Music-based Interventions (RG-MBI) Checklist—Explanation and Elaboration* (Robb et al., 2025), music-based interventions (MBIs) are defined as structured intervention programs in which musical elements constitute the core active ingredient, implemented through reproducible procedures and aimed at clearly specified health-related objectives, including music therapy, structured music training, and music listening. Prior research suggests that musical training may enhance cognitive function; for example, it can modulate synchronized cortical activity within neural networks involved in verbal memory formation (Cheung et al., 2017) and improve children's perception of speech in noise (Slater et al., 2015). However, studies examining the effects of music interventions on cognitive function in older adults have largely focused on comparisons between musicians and non-musicians (Moussard et al., 2016; Hanna-Pladdy and Gajewski, 2012; Grassi et al., 2017), or on populations with neurological disorders or mixed elderly cohorts (McCrary et al., 2022; Chiu et al., 2017; Rogers and Metzler-Baddeley, 2024). Few investigations have systematically evaluated the impact of music interventions specifically among older adults without cognitive impairment. Although one related review targeted the general older population, it combined randomized controlled trials (RCTs) with quasi-experimental designs (Zhao et al., 2021). To enhance methodological rigor, the present study comprehensively identifies and synthesizes the global literature while strictly limiting inclusion to RCTs that met standardized criteria and focusing on cognitively normal older adults. We systematically evaluate the clinical effectiveness of music-based interventions for improving cognitive function, with the aim of providing robust scientific evidence to inform clinical application in this population.

## 2 Methods

### 2.1 Literature search

A comprehensive search was conducted in the following databases: PubMed, PsycINFO, Cochrane Library, Web of Science, Embase, Scopus, CINAHL, ProQuest, and China National Knowledge Infrastructure (CNKI). The search covered each database from inception through March 1, 2025. We additionally hand-searched relevant review articles and the reference lists of all included studies to identify further records.

Using PubMed as an example (full search strategies for all databases are provided in Supplementary file Search Strategy): [(“Music Therapy” OR “Music” OR “music-based intervention” OR “music intervention^*^” OR “music therapy” OR “therapeutic music” OR “rhythmic auditory stimulation” OR “group singing” OR “choir sing^*^” OR “music listening”)] AND [(“Cognition” OR cogniti^*^ OR memory OR “executive function^*^” OR attention OR “processing speed”)] AND [(“Aged” OR “Aged, 80 and over” OR elder^*^ OR “older adult^*^” OR senior^*^ OR geriatric^*^)] AND [(randomized controlled trial OR controlled clinical trial OR random^*^ OR trial OR “clinical trial”)]. For the Chinese database (CNKI), the search string (in Chinese) was: AB=(“音乐” + “节律性听觉刺激” + “合唱” + “集体唱歌” + “音乐聆听”) and AB=(“老年” + “年长” + “高龄”) and AB=(“认知” + “记忆” + “注意” + “执行功能”).

### 2.2 Inclusion and exclusion criteria

Inclusion criteria: (1) study design: published randomized controlled trials (RCTs) with no language restrictions; (2) interventions: structured interventions in which musical elements constitute the core active component, such as music listening, music therapy, or music participation/engagement; (3) participants: older adults with intact baseline cognition (age ≥60 years; cognitively normal at baseline as determined by screening); (4) outcomes: cognitive function–related outcome measures pre-specified as endpoints (see the “Study selection and data extraction” section for details).

Exclusion criteria: (1) participants with dementia, psychiatric disorders, or other conditions associated with cognitive impairment; (2) duplicate publications arising from the same cohort; (3) studies with incomplete data reporting for which additional information could not be obtained from the study authors.

### 2.3 Study selection and data extraction

This review was conducted in accordance with the PRISMA (Preferred Reporting Items for Systematic Reviews and Meta-Analyses) 2020 guidelines (Page et al., 2021). Two reviewers independently screened full texts and extracted data based on the predefined inclusion and exclusion criteria; disagreements were adjudicated by a third, independent reviewer. For each study, we extracted the following information: basic study characteristics, participant characteristics, study design, intervention(s), comparator(s), outcome measures, and participant adherence/compliance.

Following the recommendations of the *Cochrane Handbook for Systematic Reviews of Interventions* on handling multi-arm trials (Higgins et al., 2024), if a study included multiple eligible intervention arms that contributed to the same pooled comparison, we combined the music arms into a single intervention group and compared it with the control group. When multiple control groups were available, we prioritized the no-intervention control; if unavailable, we selected a non-music active control.

Outcome measures included (Chiu et al., 2017; Li et al., 2011) global cognition, executive function, memory, and attention. Global cognition was operationalized as a composite integrating the Mini-Mental State Examination (MMSE) together with measures of executive function, memory, and attention. For memory, the preferred measure was the Wechsler Memory Scale—Logical Memory II (WMS–LM II), followed by the Verbal Learning and Memory Test (VLMT), the East Boston Memory test (immediate recall), and total Digit Span (Kowa et al., 2022; Witt et al., 2019; Dong et al., 2014). For executive function, the preferred measure was the Trail Making Test Part B (TMT-B), followed by verbal fluency—Category Switching (VF–Category Switching) and the Means–End Problem Solving test (MEPS) (Sánchez-Cubillo et al., 2009; Shao et al., 2014). For attention, the preferred measure was the Trail Making Test Part A (TMT-A), followed by the Stroop test—administered under three conditions (word, color, and word–color/interference)—and MixC_var, the mixed-cost index from a perceptual set-shifting task (Sánchez-Cubillo et al., 2009; Rubin and Meiran, 2005).

### 2.4 Assessment of study quality

We assessed outcome-level risk of bias for the included randomized controlled trials using Cochrane's Risk of Bias 2.0 (RoB 2) tool (Sterne et al., 2019). Judgments were made across the five RoB 2 domains: (1) bias arising from the randomization process; (2) bias due to deviations from intended interventions; (3) bias due to missing outcome data; (4) bias in measurement of the outcome; and (5) bias in selection of the reported result. Assessments followed the tool's signaling questions and drew on the full text, Supplementary materials, and—where available—trial registry/protocol information. For each study, outcome-level judgments were issued for the pre-specified primary cognitive outcomes (e.g., executive function, task switching). Overall risk-of-bias ratings were synthesized per RoB 2 guidance (Sterne et al., 2019): Low risk if all domains were low risk; some concerns if at least one domain raised some concerns and none were high risk; and high risk if any domain was high risk, or if multiple domains cumulatively threatened the credibility of the same outcome (e.g., ≥2 domains with some concerns). Two reviewers performed assessments independently and reached consensus through discussion; disagreements were resolved by a third reviewer.

### 2.5 Statistical analysis

We conducted a meta-analysis using a random-effects model. For continuous outcomes, we calculated mean differences (MDs) with 95% confidence intervals (CIs). When different studies used non-identical scales, we pooled standardized mean differences (SMDs) with 95% CIs. All studies meeting the inclusion criteria were incorporated into the meta-analysis; studies were neither excluded nor differentially weighted based on risk-of-bias assessments. To evaluate the robustness of the pooled estimates and the potential impact of risk of bias, we performed sensitivity analyses. Primary outcomes were analyzed according to the intention-to-treat (ITT) principle, i.e., using participants as randomized to their initial allocation. If an included study did not report a valid ITT analysis, we extracted and used the analysis method as reported in the original article. For attrition or missing data, we preferentially adopted the handling approach described by the original study. Given the general lack of follow-up data among the included trials, we extracted effect sizes at post-intervention. For studies reporting multiple assessment time points, to minimize potential confounding from differences in intervention duration and to enhance temporal consistency, we applied pre-specified rules to select the assessment closest to the mean intervention duration across all included studies for the primary analysis. Heterogeneity was assessed using Cochran's *Q* test and the *I*^2^ statistic; *I*^2^ > 50% or *p* < 0.10 indicated substantial heterogeneity. In such cases, sensitivity analyses were undertaken to explore sources of heterogeneity. All analyses were performed in RevMan 5.3, and *p* < 0.05 was considered statistically significant.

## 3 Results

### 3.1 Literature selection

**Figure 1:**
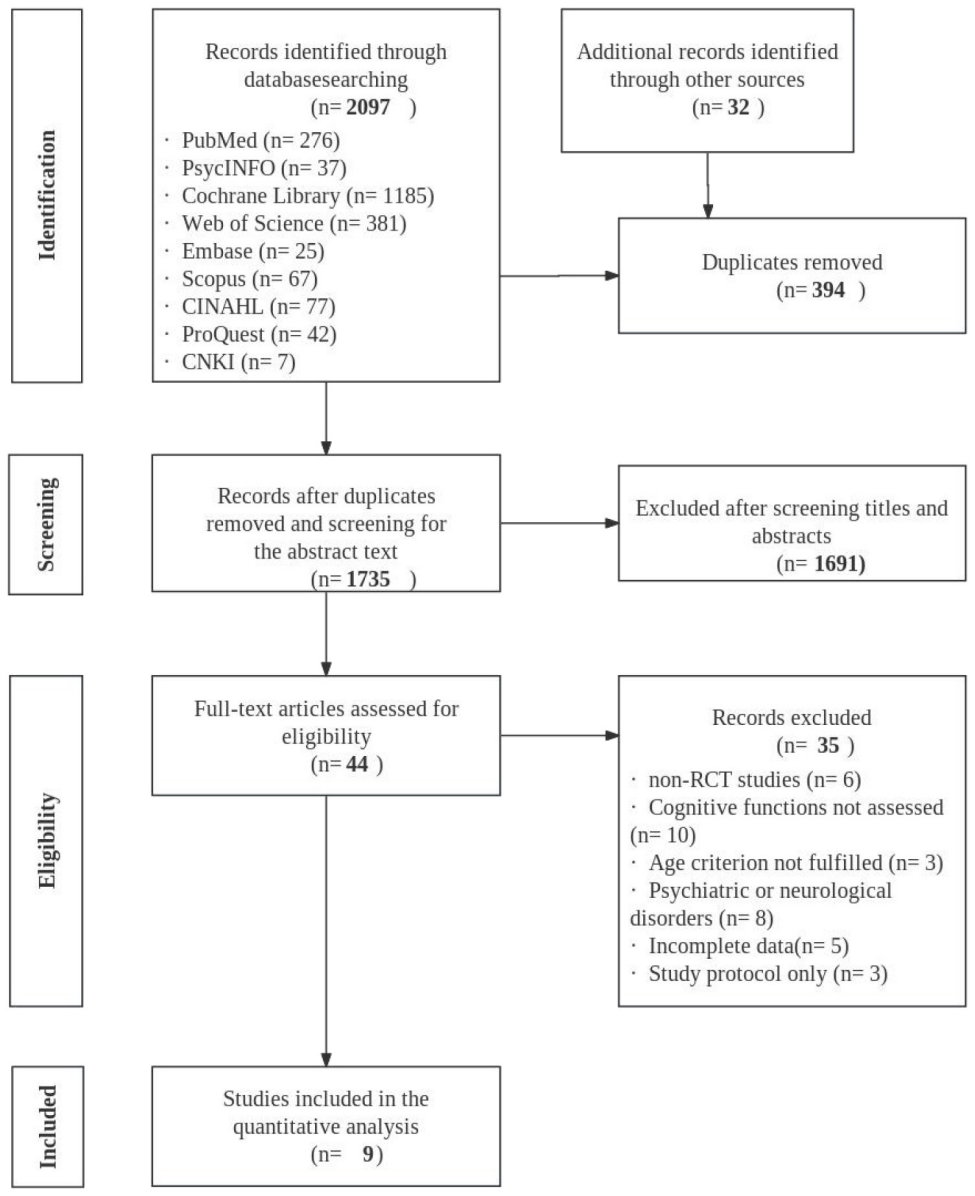
Flowchart of study identification, screening, eligibility, and inclusion.

A preliminary search identified 2,097 records. By tracing the reference lists of relevant reviews and of the included studies, 32 additional records were added manually. After removing 394 duplicates, titles and abstracts were screened and 1,691 records were excluded, leaving 44 articles for full-text assessment. Following full-text review, studies were further excluded for reasons including non-standard randomized controlled trial designs, absence of cognitive function–related measures, participants outside the target age range, enrollment of patient populations, and missing data. Ultimately, nine studies (Biasutti and Mangiacotti, 2017; Bugos et al., 2007; Bugos and Wang, 2022; Castillejos and Godoy-Izquierdo, 2020; Degé and Kerkovius, 2018; Mack et al., 2024; Marie et al., 2023; Noice and Noice, 2008; Shinada et al., 2025) were included in the systematic review. The study selection process and results are presented in Figure 1.

Nine studies were included in the systematic review, originating from the United States (*n* = 3), a dual-center study in Germany and Switzerland (*n* = 2), Germany (*n* = 1), Spain (*n* = 1), Italy (*n* = 1), and Japan (*n* = 1). The total sample comprised 625 participants, with 313 in intervention groups and 312 in control groups. All studies were randomized controlled trials. The mean intervention period was approximately 18 weeks, and the mean cumulative intervention duration across studies was 22.5 h. Detailed characteristics of the included studies are presented in Table 1.

**Table 1:** Study characteristics.

**Source**	**Country**	**Group size**		**Age, mean (SD)**		**Female (%)**		**Music intervention**		**Control condition**	**Outcomes**	**Dropout rate (%)**	
		**IG**	**CG**	**IG**	**CG**	**IG**	**CG**	**Method**	**Length**			**IG**	**CG**
Biasutti and Mangiacotti (2017)	Italy	18	17	83.39 (7.81)	83.76 (6.16)	(77.8%)	(52.9%)	Cognitive music training. 1 sessions/2 week, 70 in/session	12 weeks	TAU	MMSE; VFT; CDT; TMT-A	16.7	17.6
Bugos et al. (2007)	USA	15	16	69.6 (4.7)	71.4 (6.4)	(77.8%)	(52.9%)	Personalized piano training. 1 sessions/week, 30 min/session	24 weeks	NIC	Total Digit Span; TMT-A; TMT-B	29.4	18.8
Bugos and Wang (2022)	USA	54	50	67.2 (4.09)	67.64 (6.28)	(66.7%)	(70%)	Group sessions: Basic piano skills, repertoire, music theory. 2 sessions/week, 90 min/session	16 weeks	Wait-list	AVLT; TMT B-A; Stroop Test; VF CatSW; MEPS	11.1	28
(Castillejos and Godoy-Izquierdo 2020)	Spain	25	25	84.82 (8.08)		(76%)		Music therapy protocols. (12 group sessions + 2 individualized sessions 30–45 min/session	6 weeks	Wait-list	MMSE	0	0
Degé and Kerkovius (2018)	Germany	8	9	77.97 (2.69)	77.47 (7.08)	(100%)	(100%)	Group music activities: Singing, drumming, percussion. 1 sessions/week, 60 min/session	15 weeks	NIC	VLMT; DS-B; Symbol Sequences	0	0
Mack et al. (2024)	Switzerland and Germany	74	79	69.4 (3.15)	69.5 (3.8)	(56.2%)	(59.5%)	Group music sessions: Music theory, electronic drum, bass, keyboard performance. 1 sessions/week, 60 min/session	48 weeks	Study of music culture	PST; MixC_var	5.5	15.2
Marie et al. (2023)	Switzerland and Germany	66	66	69.2 (3.2)	69.2 (3.8)	(58%)	(59%)	Piano training. 1 sessions/week, 60 min/session	24 weeks	Study of music culture	MMSE; TWM	3	4.5
Noice and Noice (2008) ^*^	USA	40	40	82.65 (4.67)	81.6 (5.96)	(82.5%)	(90%)	Singing practice. 2 sessions/week, 60 min/session	4 weeks	NIC	DS-B; DS-F; MEPS; VFT; EBMT; WLR	9.1	14.9
Shinada et al. (2025)	Japan	13	10	69 (3.06)	69.6 (2.41)	(46.2%)	(50%)	Group piano sessions: Posture practice, score reading, rhythm exercises, music listening, performance. 1 sessions/week, 90 min/session	16 weeks	NIC	MMSE; DS-F; DS-B; TMT B-A; VFT; WMS-LM II; Stroop Test	7.1	23.1

MMSE, Mini-Mental State Examination; VFT, Verbal Fluency Test; CDT, Clock Drawing Test; TMT-A, Trail Making Test, Part A; TMT-B, Trail Making Test, Part B; AVLT, Auditory Verbal Learning Test (Rey AVLT); TMT B-A, Trail Making Test B minus A (difference score); Stroop Test, Stroop Color and Word Test; VF CatSW, Verbal Fluency (Category Switching); VLMT, Verbal Learning and Memory Test; PST, Perceptual Switch Test; MixC_var, mixed-cost index from a perceptual set-shifting task; DS-B, Digit Span—Backward; Symbol sequences, Symbol Sequences task (visuospatial working memory); DS-F, Digit Span—Forward; MEPS, Means–Ends Problem Solving; EBMT, East Boston Memory Test; WLR, Word List Recall; TWM, Tonal Working Memory; WMS-LM II, Wechsler Memory Scale Logical Memory II); IG, Intervention Group; CG, Control Group; TAU, Treatment as Usual (Usual Care); NIC, No-Intervention Control.

^*^In the study, two intervention arms were implemented—a performing-arts arm and a music arm. Because only the music arm aligned with the focus of the present review, we included only participants assigned to the music-based intervention.

### 3.2 Risk of bias assessment of included studies

Regarding the overall risk of bias, one study was rated Low risk, seven were rated some concerns, and one was rated high risk (see Table 2). Specifically, one study Noice and Noice, (2008) was judged high risk in the randomization process domain; additional issues included lack of preregistration, an open-label design, and reliance on subjective self-report scales as primary outcomes, thereby increasing the likelihood of expectancy/performance and reporting biases. For the remaining studies, the main problems clustered in the domains of deviations from intended interventions and selection of the reported result, indicating limited preregistration/analytic-plan transparency and shortcomings in reporting procedures. Two studies Bugos and Wang, (2022); Mack et al., (2024) employed intention-to-treat (ITT) analyses. Overall, the risk of bias across the included studies was predominantly some concerns, and the findings should be interpreted with caution.

**Table 2:** Risk of bias assessment of included studies.

**Study**	**Randomization process**	**Deviations from intended interventions**	**Missing outcome data**	**Measurement of outcome**	**Selection of reported result**	**Overall risk**
Biasutti and Mangiacotti (2017)	Low	Low	Low	Some concerns	Some concerns	Some concerns
Bugos et al. (2007)	Some concerns	Low	Some concerns	Some concerns	Low	Some concerns
Bugos and Wang (2022)	Low	Low	Low	Low	Low	Low
Castillejos and Godoy-Izquierdo (2020)	Some concerns	Low	Low	Some concerns	Some concerns	Some concerns
Degé and Kerkovius (2018)	Some concerns	Low	Low	Some concerns	Some concerns	Some concerns
Mack et al. (2024)	Low	Low	Some concerns	Low	Some concerns	Some concerns
Marie et al. (2023)	Low	Low	Some concerns	Low	Low	Some concerns
Noice and Noice (2008)	High	Low	Low	Some concerns	Some concerns	High
Shinada et al. (2025)	Some concerns	Low	Some concerns	Low	Some concerns	Some concerns

### 3.3 Efficacy of music-based interventions

#### 3.3.1 Efficacy of music-based interventions on global cognitive function

**Figure 2:**
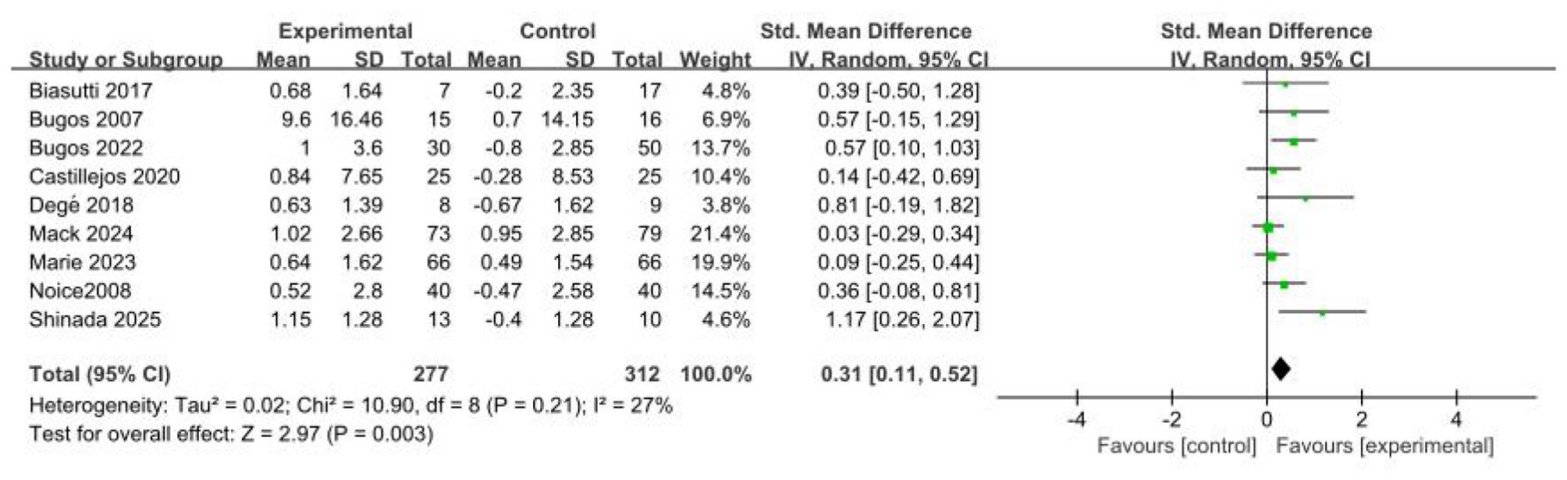
Forest plot for the efficacy of music-based interventions on global cognitive function.

Nine studies Biasutti and Mangiacotti, (2017); Bugos et al., (2007); Bugos and Wang, (2022); Castillejos and Godoy-Izquierdo, (2020); Degé and Kerkovius, (2018); Mack et al., (2024); Marie et al., (2023); Noice and Noice, (2008); Shinada et al., (2025) (*n* = 589) reported the effects of music-based interventions on global cognitive function in cognitively normal older adults. The meta-analysis showed a significant post-intervention improvement in global cognition (SMD = 0.31; 95% CI, 0.11–0.52; *p* < 0.05), with low heterogeneity (*p* = 0.21; *I*^2^ = 27%). In a sensitivity analysis excluding the trial Noice and Noice, (2008) rated high risk in the overall risk-of-bias assessment, the pooled results (RCTs = 8; *n* = 509) indicated non-significant heterogeneity (*p* = 0.15; *I*^2^ = 34%), a slightly larger point estimate with wider confidence intervals, and a persistent statistically significant difference (SMD = 0.32; 95% CI, 0.08–0.56; *p* < 0.05). To evaluate the impact of methodological heterogeneity arising from intervention type (specialist music therapy vs. general music-based interventions), we conducted a sensitivity analysis excluding the study by Castillejos and Godoy-Izquierdo (2020). The findings (RCTs = 8; *n* = 539) again showed non-significant heterogeneity (*p* = 0.15; *I*^2^ = 34%) and a statistically significant effect (SMD = 0.35; 95% CI, 0.11–0.58; *p* < 0.05). These results indicate that music-based interventions produce a significant improvement in global cognition in this population (see Figure 2).

#### 3.3.2 Efficacy of music-based interventions on memory function

**Figure 3:**
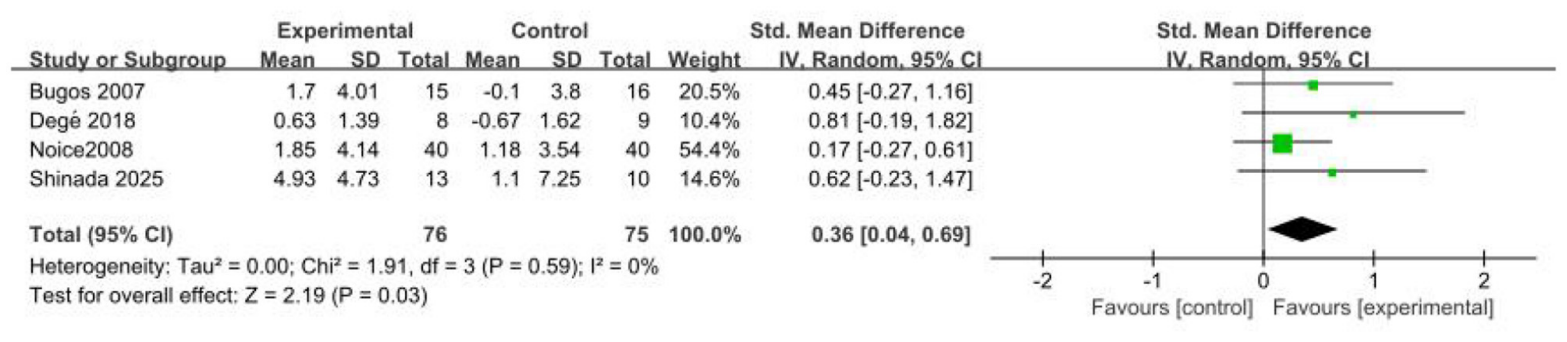
Forest plot for the efficacy of music-based interventions on memory function.

Four studies (Bugos et al., 2007; Degé and Kerkovius, 2018; Noice and Noice, 2008; Shinada et al., 2025) (*n* = 151) evaluated the effects of music-based interventions on memory function in older adults. The meta-analysis showed a significant post-intervention improvement in memory (SMD = 0.36; 95% CI, 0.04–0.69; *p* < 0.05), with no evidence of between-study heterogeneity. In a sensitivity analysis excluding the trial (Noice and Noice, 2008) rated high risk in the overall risk-of-bias assessment, the pooled results (RCTs = 3; *n* = 71) indicated non-significant heterogeneity (*p* = 0.84; *I*^2^ = 0%), a larger point estimate with wider confidence intervals, and a persistent statistically significant difference (SMD = 0.59; 95% CI, 0.11–1.07; *p* < 0.05). These findings indicate that music-based interventions yield a significant improvement in memory function in this population (see Figure 3).

#### 3.3.3 Efficacy of music-based interventions on executive function

**Figure 4:**
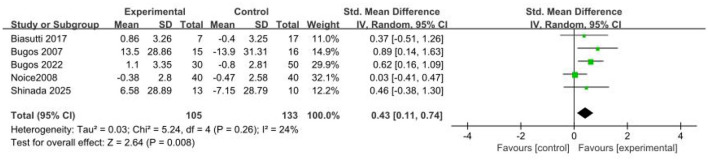
Forest plot for the efficacy of music-based interventions on executive function.

Five studies (Biasutti and Mangiacotti, 2017; Bugos et al., 2007; Bugos and Wang, 2022; Noice and Noice, 2008; Shinada et al., 2025) (*n* = 238) evaluated the effects of music-based interventions on executive function in older adults. The meta-analysis showed a significant post-intervention improvement in executive function (SMD = 0.43; 95% CI, 0.11–0.74; *p* < 0.05), with low heterogeneity (*p* = 0.26; *I*^2^ = 24%). In a sensitivity analysis excluding the trial (Noice and Noice, 2008) rated high risk in the overall risk-of-bias assessment, the pooled results (RCTs = 4; *n* = 158) indicated non-significant heterogeneity (*p* = 0.82; *I*^2^ = 0%), a larger point estimate with wider confidence intervals, and a persistent statistically significant difference (SMD = 0.61; 95% CI, 0.28–0.94; *p* < 0.05). These findings indicate that music-based interventions yield a significant improvement in executive function in this population (see Figure 4).

#### 3.3.4 Efficacy of music-based interventions on attention

**Figure 5:**
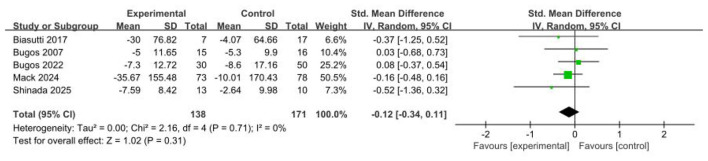
Forest plot for the efficacy of music-based interventions on attention.

Five studies (Biasutti and Mangiacotti, 2017; Bugos et al., 2007; Bugos and Wang, 2022; Mack et al., 2024; Shinada et al., 2025) (*n* = 309) evaluated the effects of music-based interventions on attention in older adults. Unlike the findings for global cognition, the meta-analysis showed no significant post-intervention effect on attention (SMD = −0.12; 95% CI, −0.34 to 0.11; *p* > 0.05), with no evidence of between-study heterogeneity. These results indicate that music-based interventions did not produce a significant improvement in attention in this population (see Figure 5).

#### 3.3.5 Publication bias

**Figure 6:**
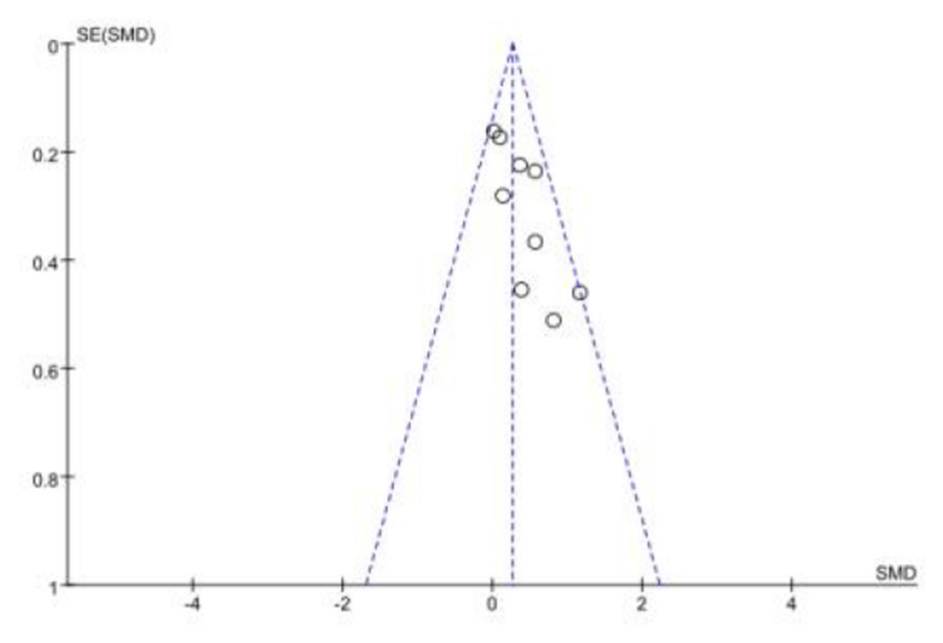
Funnel plot of publication bias.

Across the nine included studies, the funnel plot displayed an approximately symmetrical distribution. However, given the small number of studies (*n* = 9), the power of funnel-plot-based assessment is limited. Accordingly, funnel-plot asymmetry was not evident, which may suggest a low risk of substantial publication bias; nevertheless, this finding should be interpreted with caution (see Figure 6).

## 4 Discussion

This systematic review and meta-analysis synthesized nine studies to evaluate whether music-based interventions improve cognitive function in older adults. The meta-analysis indicated that music-based interventions enhanced global cognitive function in this population. When outcomes were stratified into three domains—executive function, memory, and attention—music-based interventions continued to show significant improvements in executive function and memory, whereas no significant effect was observed for attention.

### 4.1 Discussion of global cognitive function

The meta-analysis demonstrated a significant improvement in global cognitive performance among cognitively normal older adults receiving music-based interventions, consistent with our *a priori* hypothesis that such interventions enhance cognitive function. Two principal mechanisms may account for these benefits (Brancatisano et al., 2020): (1) neurobiological perspective. Music-based interventions activate widespread brain regions—including the temporal, frontal, parietal, and occipital cortices; the primary motor cortex; and subcortical structures such as the basal ganglia and the cerebellum—thereby enhancing neural flexibility and; (2) psychological perspective. Relative to other sensory stimuli, music more effectively captures attention because participants continuously track, perceive, and categorize dynamically changing features (e.g., rhythm, harmony, timbre, and meter) during intervention.

Music-based approaches can be categorized as active and passive. Active interventions require direct engagement in musical activities—such as singing, playing an instrument, or creating music—whereas passive interventions primarily involve listening to curated music to regulate psychophysiological states (Vink et al., 2004). In a 2010 study, Bugos (2010) contrasted these modalities and found that active music-based interventions markedly improved cognition in older adults, whereas passive approaches did not yield significant effects. This is broadly consistent with our findings: we observed an overall benefit for global cognition, and among the nine included trials, seven implemented active interventions (e.g., piano, electronic drums, bass, keyboard performance, singing, and drumming) and two used both modalities; studies employing active interventions accounted for >77% of the included trials. By contrast, the meta-analysis by Li et al. (2011) reported no clear cognitive improvement with active music therapy, likely because their review targeted older adults with dementia and assessed cognition solely with the Mini-Mental State Examination (MMSE). Mitchell and Malladi (2010) recommend using the MMSE in combination with additional cognitive measures to identify dementia, particularly beyond the earliest stage. In the present review, we incorporated a broader assessment battery (e.g., Digit Span, verbal fluency), which may detect changes attributable to active interventions; accordingly, active music-based engagement may positively influence global cognition in cognitively normal older adults.

### 4.2 Discussion of memory and executive function

This review further indicates that music-based interventions improve memory and executive function in cognitively normal older adults. Prior work comparing older musicians vs. non-musicians (Grassi et al., 2017) and studies in older adults with Parkinson's disease have reported similar conclusions (Thaut et al., 2009). In a study spanning multiple age groups, Jabès et al. (2021) likewise found that music-based interventions produced significant gains in working memory among older participants and explored potential links among memory capacity, aging, cognitive function, and changes in resting-state oscillatory activity. Watanabe et al. (2019) proposed two non-mutually exclusive mechanisms whereby music interventions may enhance memory in older adults: (i) when participants complete memory tasks, those experiencing a sense of task accomplishment show markedly increased neural activity in frontal and subcortical regions; and (ii) stimulation by musical melodies and verbal/lexical information may engage broader neural networks— including the basal ganglia, thalamus, and hypothalamus. These mechanisms may underlie, at least in part, the memory-enhancing effects of music interventions. Vandervert (2015) further reported that the central executive component of working memory is shaped by cortico-cerebellar system dynamics; accordingly, music interventions may strengthen central executive control and thereby augment volitional/self-regulatory control. Complementarily, Electroencephalography studies (Cheung et al., 2017) have shown that music interventions can modulate cortical synchronization within neural networks subserving verbal memory formation, which may also account for improvements in both executive function and memory.

### 4.3 Discussion of attention

This review found no significant effect of music-based interventions on attention in cognitively normal older adults. By contrast, several prior studies in healthy children have reported significant attention gains following music training (Strait et al., 2015; Dittinger et al., 2017), and extensive, long-term (multi-year) training has also improved attention in healthy adults (Strait et al., 2015; Wang et al., 2015). Three explanations may account for the null finding here. First, the music-intervention protocols used in the included trials may indeed exert little effect on attention in cognitively normal older adults; across the nine trials in the meta-analysis, the mean intervention duration was 18 weeks, and only one study (Mack et al., 2024) exceeded 6 months—importantly, that 1-year intervention did observe significant improvements in attention-related outcomes. Second, the small number of attention measures and limited sample sizes may have yielded small estimated effects and insufficient statistical power. Third, the attention measures employed may have been insufficiently sensitive to detect training-related changes in older adults. In the included literature, attention was assessed mainly with the Trail Making Test (TMT) or Digit Span Forward (DSF); drawing on additional tasks from the broader literature could improve detection. For example, Strait et al. (2015) asked participants to identify—and act out—one of two simultaneously presented stories delivered from left and right loudspeakers; Nan et al. (2018) used a masked-word target recognition task and reported enhanced executive attention following piano training in children. Consequently, future studies might increase sensitivity to change in older adults by combining multiple attention measures within a comprehensive assessment battery.

### 4.4 Limitations and future research directions

This study has several limitations. First, the available evidence is limited in quantity and lacks cultural and geographic representativeness. Trials of music-based interventions targeting cognitively normal older adults remain few, are mostly conducted in high-income countries, and focus predominantly on Western instrumental training (e.g., piano, percussion). There is insufficient attention to traditional musical practices representative of developing countries such as China (e.g., guqin, Chinese opera), constraining external validity and cultural applicability. Second, the nature of music interventions makes blinding difficult, introducing risks of performance and detection bias. Blinding participants and interventionists is typically infeasible; although some studies attempted to blind outcome assessors, most did not fully mitigate expectancy effects among participants. In addition, sample sizes were generally small: among the nine included studies, five enrolled fewer than 50 participants, which may reduce statistical power to detect small effects and may also inflate effect-size estimates. Finally, there was heterogeneity in intervention protocols. Although all interventions were music-centered, their specific formats, intensities, frequencies, and durations varied, potentially increasing between-study heterogeneity and complicating the identification of the most effective intervention model.

Based on this systematic review, we propose several recommendations and considerations for future research. First, while continuing to address the needs of older adults with cognitive impairment, future work should concurrently pursue preventive trials among those without overt cognitive decline to slow cognitive aging. Parallel studies in developing countries are particularly valuable to test cross-cultural generalizability and improve the representativeness of study samples. Second, methodological rigor should be strengthened by implementing robust allocation concealment; blinding outcome assessors; and adopting preregistered protocols with prespecified statistical analysis plans. Finally, factorial or adaptive designs should be used to compare different modalities (e.g., instrumental training, rhythmic interventions, choir/singing, receptive listening) and different doses (e.g., per-session duration, frequency, total hours), in order to identify the active ingredients that drive benefit (e.g., motor engagement, cognitive load, social participation).

## 5 Conclusion

This systematic review and meta-analysis indicates that among cognitively normal older adults, music-based interventions significantly enhance global cognition, executive function, and memory, whereas their effect on attention was not statistically significant. Given the limited sample sizes of the included trials, these findings warrant confirmation in larger, high-quality studies.

## Data Availability

The original contributions presented in the study are included in the article/Supplementary material, further inquiries can be directed to the corresponding author.
