# Supplementary material for: The effects of music-based interventions on cognitive function in cognitively normal older adults: a systematic review and meta-analysis
Source: Front Psychol. 2025 Nov 18;16:1632873. doi: 10.3389/fpsyg.2025.1632873 (PMC12669183; doi:10.3389/fpsyg.2025.1632873)
Supplement: Supplementary file 1 [file Data_Sheet_1.pdf]

Search strategies

1. PubMed

("Music Therapy"[Mesh] OR "Music"[Mesh] OR music*[tiab] OR "music-based"[tiab] OR "music-based intervention"[tiab] OR "music intervention*"[tiab] OR "music therapy"[tiab] OR "therapeutic music"[tiab] OR "rhythmic auditory stimulation"[tiab] OR "group singing"[tiab] OR "choir sing*"[tiab] OR "music listening"[tiab])
AND
("Cognition"[Mesh] OR cogniti*[tiab] OR memory[tiab] OR "executive function*"[tiab] OR attention[tiab] OR "processing speed"[tiab])
AND
("Aged"[Mesh] OR "Aged, 80 and over"[Mesh] OR elder*[tiab] OR "older adult*"[tiab] OR senior*[tiab] OR geriatric*[tiab])
AND
(randomized controlled trial[pt] OR controlled clinical trial[pt] OR random*[tiab] OR trial[tiab] OR "clinical trial"[Publication Type])

2. APA PsycINFO

((TI(music*) OR AB(music*) OR TI("music therapy") OR AB("music therapy") OR TI("music-based intervention") OR AB("music-based intervention") OR TI("rhythmic auditory stimulation") OR AB("rhythmic auditory stimulation") OR TI("group singing") OR AB("group singing") OR TI("choir sing*") OR AB("choir sing*") OR TI("music listening") OR AB("music listening")))
AND
(TI(cogniti* OR memory OR "executive function*" OR attention OR "processing speed") OR AB(cogniti* OR memory OR "executive function*" OR attention OR "processing speed"))
AND
(TI(elder* OR "older adult*" OR senior* OR geriatric*) OR AB(elder* OR "older adult*" OR senior* OR geriatric*))
AND
(PT("Randomized Controlled Trial") OR PT("Clinical Trial") OR AB(random* OR trial) OR TI(random* OR trial))

3. Cochrane Library

("Music Therapy" OR "Music" OR "music-based intervention" OR "rhythmic auditory stimulation" OR "group singing" OR "choir sing*" OR "music listening")
AND
("Cognition" OR cogniti* OR memory OR "executive function*" OR attention OR "processing speed")
AND
("Aged" OR "older adult*" OR elder* OR senior* OR geriatric*)

4. **Web of Science**

TS=(("music therapy" OR "music-based intervention" OR "rhythmic auditory stimulation" OR "group singing" OR "choir sing*" OR "music listening" OR music*) AND ("cogniti*" OR memory OR "executive function*" OR attention OR "processing speed") AND ("older adult*" OR elder* OR senior* OR geriatric*) AND (random* OR trial))

5. **Embase**

('music therapy'/exp OR 'music'/exp OR music:ti,ab,kw OR 'music therapy':ti,ab,kw OR 'music-based intervention':ti,ab,kw OR 'rhythmic auditory stimulation':ti,ab,kw OR 'group singing':ti,ab,kw OR 'choir sing*':ti,ab,kw OR 'music listening':ti,ab,kw)
AND
('cognition'/exp OR cogniti*:ti,ab,kw OR memory:ti,ab,kw OR 'executive function*':ti,ab,kw OR attention:ti,ab,kw OR 'processing speed':ti,ab,kw)
AND
('aged'/exp OR elderly:ti,ab,kw OR 'older adult*':ti,ab,kw OR senior*:ti,ab,kw OR geriatric*:ti,ab,kw)
AND
('randomized controlled trial'/exp OR 'clinical trial'/exp OR random*:ti,ab,kw OR trial:ti,ab,kw)

6. **Scopus**

TITLE-ABS-KEY(("music therapy" OR "music-based intervention" OR "rhythmic auditory stimulation" OR "group singing" OR "choir sing*" OR "music listening" OR music*) AND ("cogniti*" OR memory OR "executive function*" OR attention OR "processing speed") AND ("older adult*" OR elder* OR senior* OR geriatric*) AND (random* OR trial))

7. **CINAHL**

(MH "Music Therapy" OR TI("music therapy" OR "music-based intervention" OR "rhythmic auditory stimulation" OR "group singing" OR "choir sing*" OR "music listening" OR music*) OR AB("music therapy" OR "music-based intervention" OR "rhythmic auditory stimulation" OR "group singing" OR "choir sing*" OR "music listening" OR music*))
AND
(MH "Cognition" OR TI(cogniti* OR memory OR "executive function*" OR attention OR "processing speed") OR AB(cogniti* OR memory OR "executive function*" OR attention OR "processing speed"))
AND
(MH "Aged+" OR TI("older adult*" OR elder* OR senior* OR geriatric*) OR AB("older adult*" OR elder* OR senior* OR geriatric*))
AND
(PT "Randomized Controlled Trial" OR PT "Clinical Trial" OR TI(random* OR trial) OR AB(random* OR trial))

8. **ProQuest**

NOFT("music therapy" OR "music-based intervention" OR "rhythmic auditory stimulation" OR "group singing" OR "choir sing*" OR "music listening" OR music*)
AND NOFT(cogniti* OR memory OR "executive function*" OR attention OR "processing speed")
AND NOFT("older adult*" OR elder* OR senior* OR geriatric*)
AND NOFT(random* OR trial)

9. **CNKI**

AB=("音乐"+"节律性听觉刺激"+"合唱"+"集体唱歌"+"音乐聆听")
AND
AB=("老年"+"年长"+"高龄")
AND
AB=("认知"+"记忆"+"注意"+"执行功能")
